# Case report: Spontaneous closure of ventricular pseudoaneurysm post-acute myocardial infarction with non-surgical therapy

**DOI:** 10.3389/fcvm.2022.996072

**Published:** 2022-09-20

**Authors:** Xinxin Shuai, Xiajun Hu, Yumiao Wei

**Affiliations:** Department of Cardiology, Union Hospital, Tongji Medical College, Huazhong University of Science and Technology, Wuhan, China

**Keywords:** ventricular pseudoaneurysm, myocardial infarction, shape, hemodynamic, conservative therapy, outcomes

## Abstract

Left ventricle (LV) pseudoaneurysm is a rare disorder post-acute myocardial infarction (AMI). Resection or closure of the pseudoaneurysm by surgery is recommended due to the high propensity of pseudoaneurysm rupture while surgery has also high risks. Conservative therapy could be acceptable in small pseudoaneurysms or patients with high surgical risks. Nevertheless, the risk evaluation and grasp of indication are not clear. This case reported an acute cyst-like LV pseudoaneurysm formation post-AMI-induced myocardial free wall rupture (MFWR), and the patient recovered with spontaneous closure of the fissure and shrinkage of the LV pseudoaneurysm through non-surgical therapy. Based on the observations in the echocardiogram, we proposed that intermittent closing of the fissure and interruption of the blood flow between the LV and the pseudoaneurysm due to LV contraction alleviated stress change on the pseudoaneurysm. The narrow fissure, small pseudoaneurysm, and intermittently interrupted blood flow that benefit fissure healing and pseudoaneurysm stabilization could indicate the prognosis of this patient. Drugs like β-blocker that decreased the stress on the pseudoaneurysm also led to the risk reduction of pseudoaneurysm rupture. To our knowledge, this is the first case that reports a spontaneous closure of LV pseudoaneurysm. The size of the fissure and the pseudoaneurysm, as well as the corresponding hemodynamic state, could be valuable to evaluate the risk and prognosis of the pseudoaneurysm. Optimized medical management was also helpful to pseudoaneurysm stabilization.

## Introduction

Left ventricle (LV) pseudoaneurysm formation is a rare complication of acute myocardial infarction (AMI) with a poor prognosis (1). It is a consequence of myocardial free wall rupture (MFWR), followed by pericardial adhesions, enclosing the fissure to form a fibrous chamber, and keeping interflow with the ventricular chamber. Pseudoaneurysm commonly occurs in the posterior or lateral wall of LV, with an incidence of 0.1% in MI (2). A pseudoaneurysm is more dangerous than a true aneurysm because of the high propensity to rupture and sudden death (3). Other symptoms could be congestive heart failure, arrhythmia, or thrombosis. Some patients could be asymptomatic and the illness can be discovered by imaging examination. Pseudoaneurysm resection or closure through surgical intervention (3) is the major therapeutic strategy; meanwhile, conservative therapy is also observed in some cases. However, a detailed information about risk evaluation, strategy making, long-term management, and associated outcomes is not clear. Here, we firstly reported an acute cyst-like LV pseudoaneurysm formation post-AMI; the patient recovered with spontaneous closure of the fissure and shrinkage of the pseudoaneurysm through non-surgical therapy. The width of the fissure that determines whether the fissure could be closed, and the blood flow could be interrupted during myocardial contraction could be an important parameter to evaluate the pseudoaneurysm risk and prognosis of the patient. The case will be presented in accordance with the CARE reporting checklist.

## Case description

**Figure 1:**
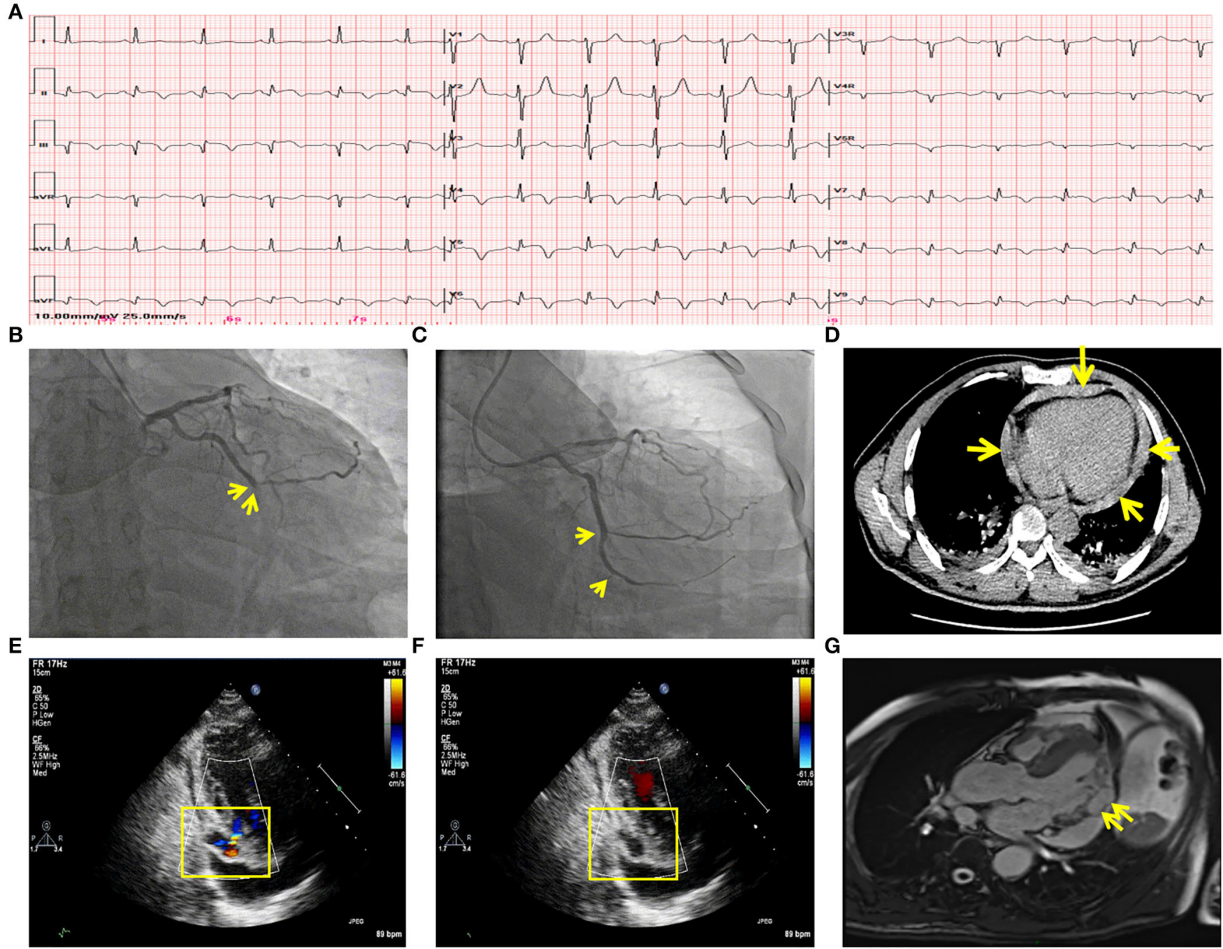
Clinical data of the patient with LV pseudoaneurysm post-acute myocardial infarction. **(A)** ECG showed acute inferior-, posterior- and lateral wall myocardial infarction. **(B)** Coronary angiography showed LCX acute occlusion. Yellow arrows indicated the occluded point of the LCX. **(C)** The primary percutaneous coronary intervention of LCX. A yellow arrow marked the revascularization of the proximal segment of the LCX. **(D)** CT demonstrated hemopericardium which was marked by yellow arrows. **(E)** TTE showed an LV pseudoaneurysm formation and blood between the pseudoaneurysm and LV during the diastolic period. **(F)** TTE showed blood flow interruption during the systolic period. **(G)** CMR confirmed LV pseudoaneurysm formation in the lateral wall, which is marked by the yellow arrow. LV, left ventricle; ECG, electrocardiogram; LCX, left circumflex; TTE, transthoracic echocardiography; CMR, cardiac magnetic resonance imaging.

**Figure 2:**
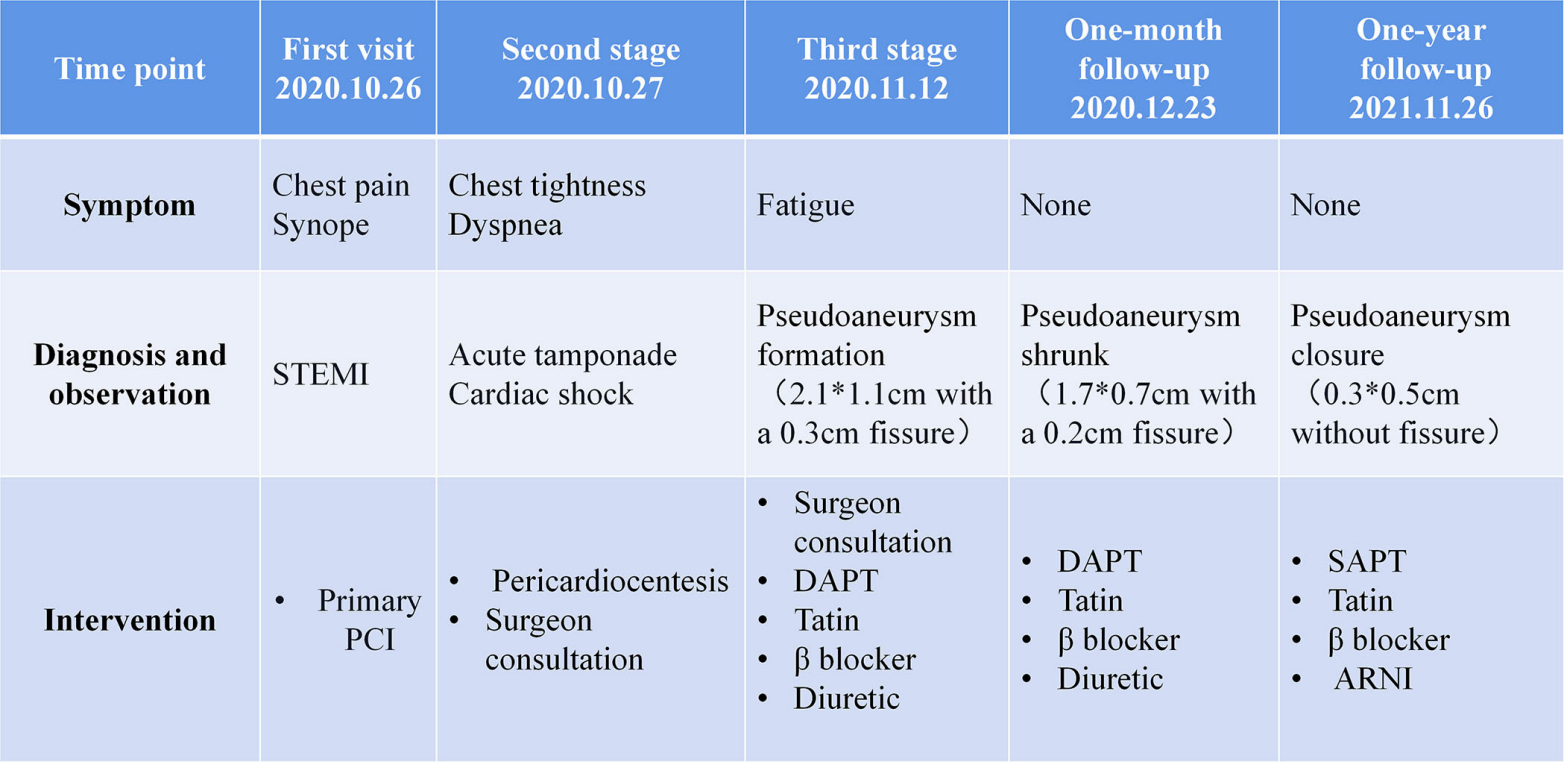
Clinical diagnosis and treatment timeline of the patient. STEMI, ST-segment elevation myocardial infarction; PCI, percutaneous coronary intervention; DAPT, dual-antiplatelet therapy; SAPT, single antiplatelet therapy; ARNI, angiotensin receptor, and neprilysin inhibitor.

A 53-year-old male patient was admitted to CCU because of sudden syncope for 8 h, which was preceded by intermittent chest pain for 3 days, while the patient did not seek medical attention. He had a history of hypertension and diabetes mellitus for 2 years without medical intervention, his home blood pressure (BP) was about 160/80 mmHg, and his blood glucose was not monitored. He also had a smoking history of 20 years. No family history or psychosocial history was declared. The electrocardiogram (ECG) of the patient showed ST segment elevation in leads II, III, aVF, V5-V6, and V7-V9, which suggested inferior-, posterior-, and lateral-wall AMI (Figure 1A). Primary percutaneous coronary intervention (PCI) of the occlusive proximal segment of the left circumflex (LCX) coronary artery (Figure 1B) was successfully performed (Figure 1C). The blood pressure of the patient was 96/60 mmHg, and the resting heart rate (HR) was 118 beats per minute (bpm). He had impaired hepatic function, with alanine aminotransferase (ALT) raised to 3,751 U/L (normal < 40 U/L), and aspartate aminotransferase (AST) elevated to 6,804 U/L (normal < 40 U/L). Creatinine was elevated to 393.8 μmol/L (normal 44–133 μmol/L). Arterial blood gas analysis showed decreased pH level (7.27) and elevated lactate level (5.7 mmol/L). The patient performed typical symptoms of shock that cannot be attributed to simple LCX proximal segment occlusion. A bedside transthoracic echocardiography (TTE) was urgently performed and found mild pericardial effusion. Chest CT (CT) further confirmed that the pericardium effusion was bloody (Figure 1D). Subacute cardiac rupture and pericardial tamponade were highly suspected. Emergency pericardiocentesis and drainage of bloody effusion were subsequently performed. The BP of the patient rapidly raised to 168/90 mmHg, the HR reduced to 90-100 bpm, and shock-associated symptoms were also relieved. A cardiac surgeon was called for an emergency consultation. The surgical department estimated that surgery was recommended, while the postoperative risk was high in this patient. After adequate notification of the therapeutic strategies and associated prognosis, the patient and his family finally chose conservative intervention instead of surgery. However, subsequent TTE reexamination revealed the presence of a 2.1^*^1.1 cm pseudoaneurysm with a width of 0.3 cm fissure in the lateral wall that connected the LV and the pseudoaneurysm (Figures 1E,F), while cardiac magnetic resonance (CMR) also demonstrated the LV pseudoaneurysm formation (Figure 1G). Interestingly, during diastole, the blood flowed between the LV and pseudoaneurysm (Figure 1E, Supplementary Video 1); while during systole, the fissure was closed due to myocardial contraction, and the blood flow was subsequently interrupted (Figure 1F). Comprehensively considering the risk of the disease and the willingness of the patient, we carried out a conservative strategy. Aspirin and ticagrelor were continuously prescribed. Betaloc was also used in sufficient doses, aiming to decrease the stress on the fissure and the pseudoaneurysm. A low dose of diuretics was reserved to gently reduce volume overload. Twenty-eight days later, the patient was discharged without any symptoms, such as dyspnea or chest pain. The pre-discharge TTE still observed a 1.5^*^1. pseudoaneurysm with a 0.3 cm-wide fissure, and the ejection fraction of LV was 55%. One month after discharge, the patient came for a follow-up. TTE showed that the LV pseudoaneurysm was reduced to 1.7^*^.7 cm, and the fissure was 0.2 cm. Mild pericardial effusion was still observed without any symptoms. One year later, TTE reexamination revealed that the LV pseudoaneurysm was only about 0.3^*^0.5 cm, and the fissure was closed without blood flow between the ventricle and the pseudoaneurysm (Supplementary Figure 1, Supplementary Video 2). Meanwhile, pericardial effusion was not observed anymore. The clinical diagnosis and treatment timeline of the patient was summarized in Figure 2.

## Discussion

Pseudoaneurysm is fatal because of the risk of rupture. According to previous literature (Table 1), most patients accepted surgical therapy. The outcomes were diverse in different clinical centers, operation associated mortality ranged from 0 to 35.7% (1–3, 5–11), mortality during follow-up ranged from 0 to 31% (1–6, 8–11), and patients with acute pseudoaneurysm or high surgical risks had higher mortality (3). The mortality of conservative therapy was also discussed in some studies with a small sample size, which appeared to be higher than surgical therapy (1, 4, 5, 7, 11). Meanwhile, some retrospective studies reported patients with chronic small (< 3 cm in size) LV pseudoaneurysm or patients with high surgical risks could be managed conservatively (12). Based on these data, surgery should be the first choice for patients with pseudoaneurysms, while conservative therapy could be acceptable in some special situations. The question is how to evaluate the risk and prognosis for each patient. Our patient surprisingly underwent a spontaneous closure of the pseudoaneurysm without surgery, which was not reported since. Several case reports also gave detailed information about patients with non-surgical therapy (13–15), while all these pseudoaneurysms kept the same or expanded size. The factors that participated in the closure of pseudoaneurysm in our case is worthy of discussion. Since ventricular anatomy is important in maintaining cardiac function (16), the anatomic and corresponding hemodynamic change due to pseudoaneurysm formation in the LV could give some indications.

**Table 1:** Summary of clinical investigations about LV pseudoaneurysm.

**Ref/Year**	**Size of** **pseudo-aneurysm (cm)**	**Patients with surgery**	**Operation related mortality**	**Follow up mortality in surgical patients**	**Patients with conservative therapy**	**Follow up mortality in conservative patients**
Frances et al. (4)/1998	6.0 (1.5, 20.1)	193	/	23%	31	48%
Tiong et al. (5)/1998	/	42	7%	31%	10	60%
						No cardiac rupture
Yeo et al. (1)/1999	4.53 ± 2.03	16	13%	50%	6	83%
						No cardiac rupture
Prêtre et al. (6)/2000	/	10	30%	30%	/	/
Moreno et al. (7)/2003	9.8 ± 6.9	1	0%	0%	10	20%
						No cardiac rupture
Lafci (8)/2006	/	8	12.5%	1 CHF	/	/
Eren et al. (2)/2007	4.7 ± 0.48	14	35.7%	1 sudden death	/	/
				1 cancer		
Fernando et al. (9)/2007	/	30	20%	27%	/	/
Narin et al. (10)/2008	/	5	0%	0%	/	/
Prifti et al. (3)/2017	4.2 ± 0.7	12	30.8%	15%	/	/
Zhong et al. (11)/2022	/	10	0%	0%	7	42.9%

Ref, reference; CHF, chronic heart failure.

**Figure 3:**
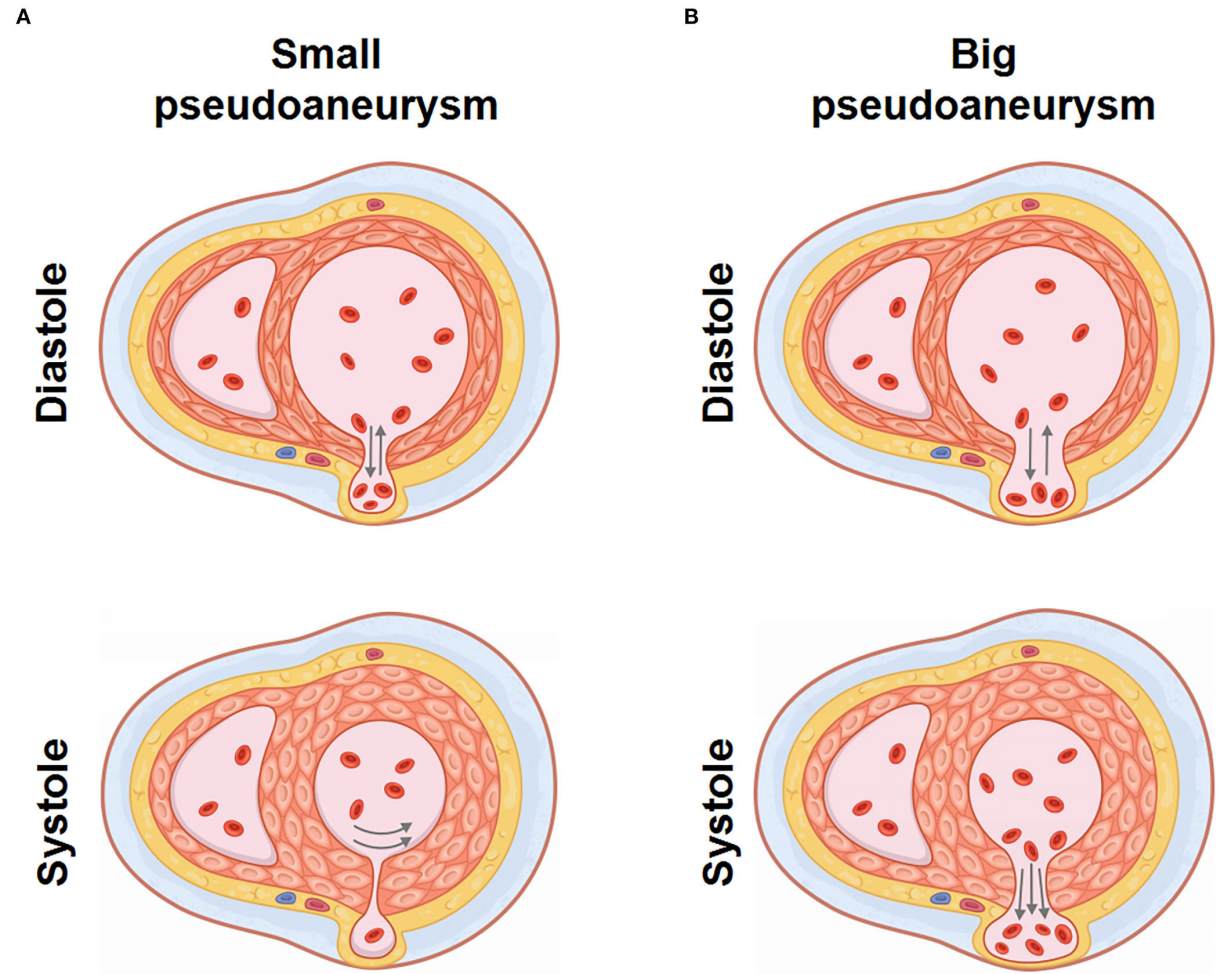
Hemodynamic characteristics of LV pseudoaneurysm with different shapes. **(A)** Hemodynamic diagram of small LV pseudoaneurysm with narrow fissure. **(B)** Hemodynamic diagram of big LV pseudoaneurysm with wide fissure.

Pseudoaneurysm keeps blood flow with LV, bearing stress from the ventricular chamber and pericardial cavity. With the movement of LV, altering stress could result in pseudoaneurysm expansion or even rupture. Through TTE and CMR, we observed a specific cardiac movement pattern in our patient with a small pseudoaneurysm, which is dissimilar from a big pseudoaneurysm that continuously keeps a connection with LV (17, 18). We made a schematic diagram to illustrate the hemodynamic state. In a small pseudoaneurysm with a narrow fissure, the fissure relaxes and blood flows between LV and the pseudoaneurysm during the ventricle diastole. In this period, LV pressure is low, and stress endured by the pseudoaneurysm is small. Meanwhile, the pressure from the pericardial due to the presence of effusion provides centripetal force in the pseudoaneurysm, offsetting the pressure from the ventricle. During cardiac systole, the fissure is tightened because of ventricular wall contraction, resulting in a temporary partition between the pseudoaneurysm and the LV. Pressure in the pseudoaneurysm was not influenced by sharply increased LV pressure (Figure 3A, Supplementary Video 1). As with a big pseudoaneurysm with a wide fissure, the pseudoaneurysm keeps blood transfer with LV, the stress and shape change are continuously influenced by the changing LV pressure (Figure 3B), resulting in worse cardiac function and pseudoaneurysm instability. Therefore, we conclude that for small pseudoaneurysms with narrow fissures, LV contraction tends to promote the rupture healing by tightening the fissure, and patients with conservative therapy may survive under this condition. While a big pseudoaneurysm keeps consistent and unstable blood flow with LV, conservative management is associated with higher mortality. This is an uncommon case that reported a spontaneous closure of ventricular pseudoaneurysm, while similar cases or clinical studies are not retrieved. Torchio et al. also recently mentioned that the size of the pseudoaneurysm could partly determine the intervention strategy (19), while due to the limited case, further observations are needed to promote our cognition.

Concerning to medical therapy, long-term medication of ACEI/ARB or β-blocker was observed in some conservative cases (13–15). Some studies retrospected clinical characteristics of MFWR and a summarized higher ACEI/ARB and β-blocker coverage was associated with lower in-hospital incidence and mortality (20). Nonetheless, there is no evidence-based medicine supporting the efficiency in pseudoaneurysm formation post-MFWR. For patients who suffered acute pseudoaneurysms without surgical therapy, promoting rupture repair could be more important. ACEI/ARB was avoided in the early stage because of their inhibition of blood pressure and cardiac fibrosis. Oral β blocker is prescribed because β-blocker can block catecholamine adrenergic receptors-mediated cardiotoxicity in MI. In this patient, β-blocker prolonged ventricular diastolic time to increase returned blood volume. On the other hand, the negative inotropic effect reduced the myocardial stretch on the fissure and the pseudoaneurysm. Through these methods, the pseudoaneurysm was placed in a relatively stable mechanical environment, which reduced the risk of rupture and strove for the opportunity of myocardial wound healing.

In some cases, or small retrospective analysis, mechanical circulatory support (MCS) could be beneficial in stabilizing the hemodynamic conditions in patients with mechanical complications post-AMI and building a bridge to a safer timing of surgery. For example, La Torre et al. (21) described their first experience in the use of Impella in patients with cardiogenic shock due to acute ventricular septal defect (VSD) post-AMI. Patanè et al. (22) also confirmed the advantages of Impella in improving patients' hemodynamic state and survival rate. Extracorporeal membrane oxygenation (ECMO) support in patients with MFWR was also reported as a single case (23, 24), although the anticoagulation strategy was not clearly described. Compared with Impella and ECMO, Intraaortic balloon counterpulsation (IABP) is a more available device. Even though IABP-SHOCK trials concluded that IABP did not reduce short and long-term mortality in patients with cardiogenic shock (25, 26), IABP is still commonly used in acute VSD post-AMI, while the usage in patients with MFWR or LV pseudoaneurysm was not reported (27). Meanwhile, the adverse effects of mechanical device implantation should be noticed. Further trials are needed to optimize the MCS strategy. In our patient, hemodynamic instability was rapidly relieved after pericardiocentesis, we evaluated that MCS was unnecessary after weighing the benefits and risks of this approach.

## Conclusion

This is the first case report of spontaneous closure of a pseudoaneurysm post AMI with non-surgical therapy. The shape and movement pattern of the pseudoaneurysm could promote rupture healing. Careful conservative management based on stress control and wound healing synergistically facilitated the healing process. This case highlights the importance of shape characteristics and hemodynamic state of a pseudoaneurysm in the risk and prognosis evaluation. With the limitation of the number of cases, whether the experience can be expanded or even provide information for clinical decision need to be further investigated.

## Data availability statement

The raw data supporting the conclusions of this article will be made available by the authors, without undue reservation.

## Ethics statement

Written informed consent was obtained from the individual(s) for the publication of any potentially identifiable images or data included in this article.

## Author contributions

XS designed the case report, analyzed the patient data, and revised the manuscript. XH analyzed the patient data and drafted the manuscript. YW designed the case report and collected the patient data. All authors read and approved the final manuscript.

## Funding

This work was supported by the National Natural Science Foundation of China (Grant Nos. 31901004 and 82070376).

## Conflict of interest

The authors declare that the research was conducted in the absence of any commercial or financial relationships that could be construed as a potential conflict of interest.

## Publisher's note

All claims expressed in this article are solely those of the authors and do not necessarily represent those of their affiliated organizations, or those of the publisher, the editors and the reviewers. Any product that may be evaluated in this article, or claim that may be made by its manufacturer, is not guaranteed or endorsed by the publisher.
