# Supplementary figures and images for: Case report: Spontaneous closure of ventricular pseudoaneurysm post-acute myocardial infarction with non-surgical therapy
Source: Front Cardiovasc Med. 2022 Sep 20;9:996072. doi: 10.3389/fcvm.2022.996072 (PMC9530630; doi:10.3389/fcvm.2022.996072)

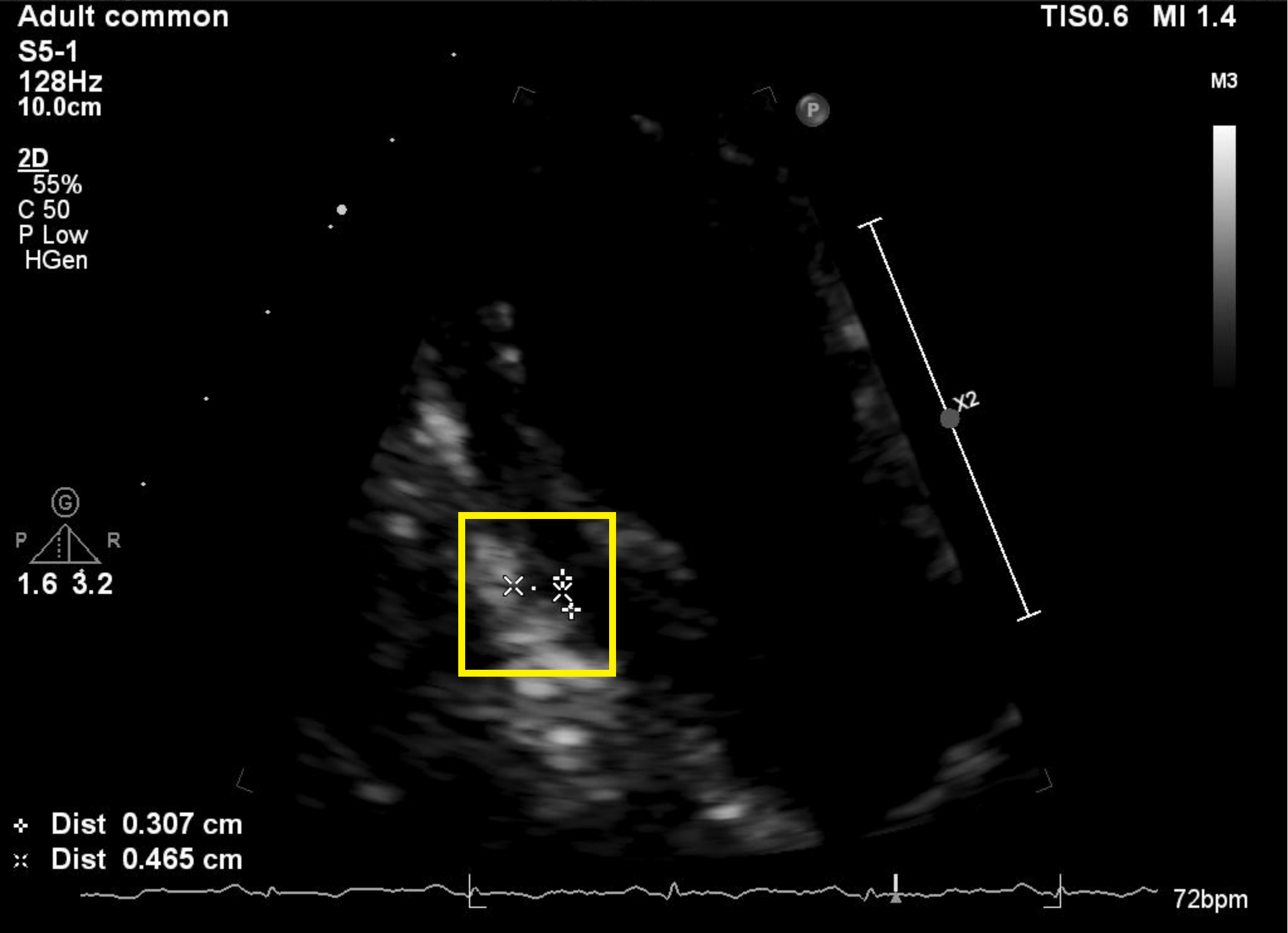

Supplement: Supplementary file 3 [file Image_1.TIFF]
